# From the Seat of War!

**Published:** 1855-07

**Authors:** 


*From the Seat of War!*—Dr. Reese, of the American Medical Gazette, is suffering under an attack of libel suit, inflicted by Prof. Draper, of the University of New York. Prof. Draper claims to have been damnified (that's the legal term, as we know from the perusal of several sheets of foolscap, politely handed us by an embryo lawyer a few weeks since) in a considerable sum, by Dr. Reese's attacks upon him as a man and teacher.

Now we hope that our independence is a fixed fact, and that when we say that we look upon libel suits as unholy attacks upon the freedom of the press, we shall not—a la Peninsular—be accused of personal motives, simply because we are so fortunate as to have an affair of the kind ourselves. Indeed we have nothing to say about this latter circumstance. We shall watch the contest with a due share of anxiety, and shall inform our readers who is elected, immediately after the polls close. In the meantime, however, we shall console ourselves with the misfortunes of others. And among the most unfortunate of these is Prof. Draper himself. With all the differences among the editorial fraternity they have their share of *esprit du corps*. The news of this libel suit was the signal for a combined attack on the University of New York, by medical journals north, south, east, and west. Dr. Reese has no editorial enemy who is virulent enough to side against him now, while the weak points of the University, its bragging, its pretention, its egotism, and the thousand humbugs on which it has subsisted for the past few years, are brought to the light in a most unmerciful manner.

We cannot but regard the action of Prof. Draper as singularly ill-timed. The school over which he presides had just associated with its faculty some men of talent, learning, and probity, good men and good teachers. The recoil of this libel suit will involve them as well as Dr. Draper.

